# Supplementary figures and images for: An amplicon-based nanopore sequencing workflow for rapid tracking of avian influenza outbreaks, France, 2020-2022
Source: Front Cell Infect Microbiol. 2024 Jan 22;14:1257586. doi: 10.3389/fcimb.2024.1257586 (PMC10839014; doi:10.3389/fcimb.2024.1257586)

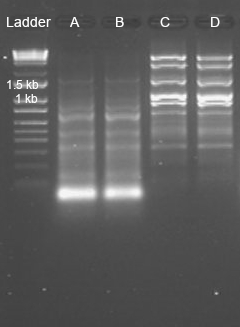

Supplement: Supplementary file 1 [file Image_1.jpeg]

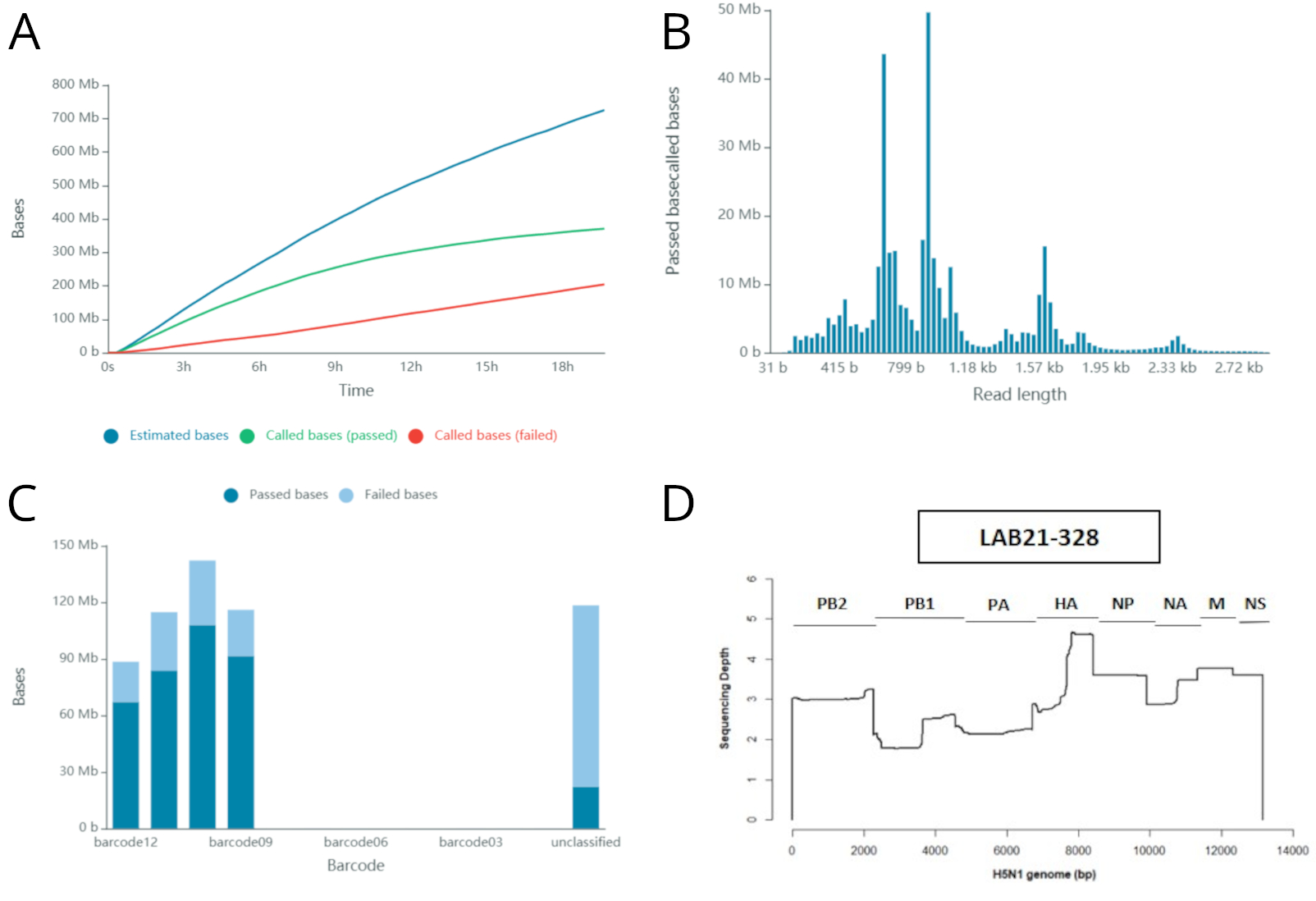

Supplement: Supplementary file 2 [file Image_2.jpeg]

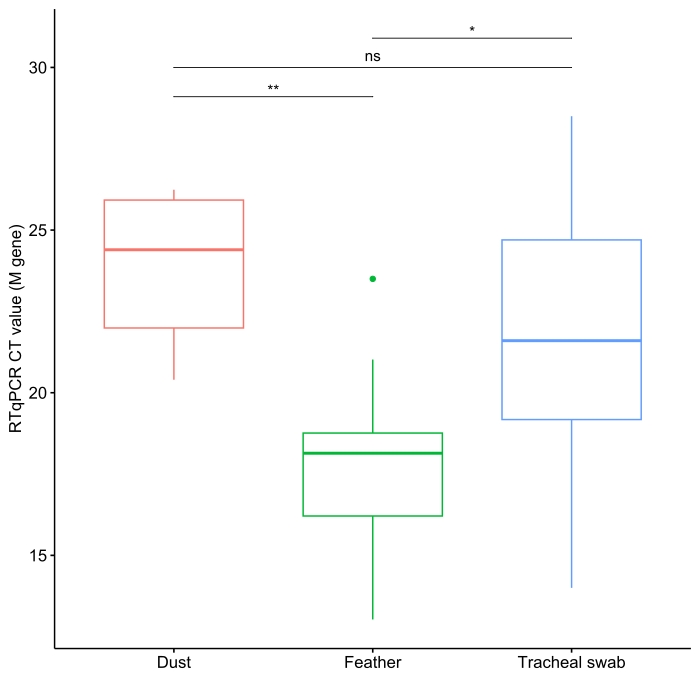

Supplement: Supplementary file 3 [file Image_3.jpeg]

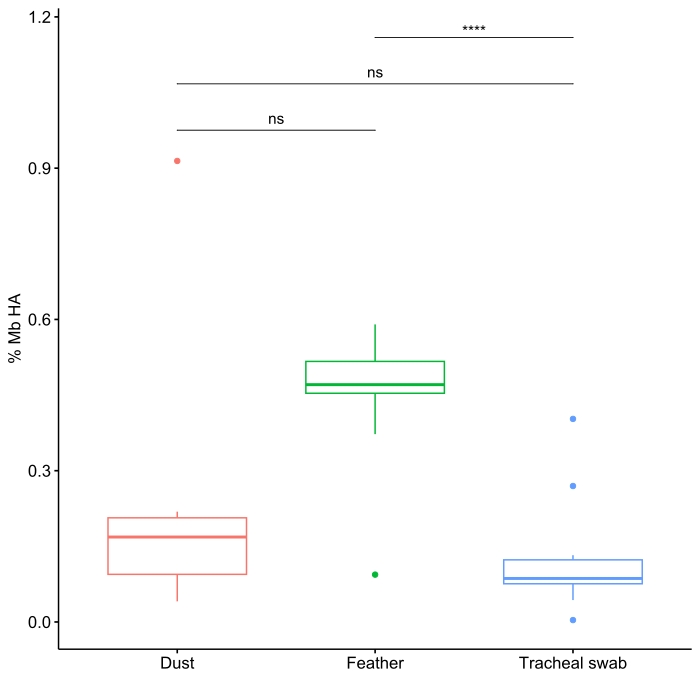

Supplement: Supplementary file 4 [file Image_4.jpeg]

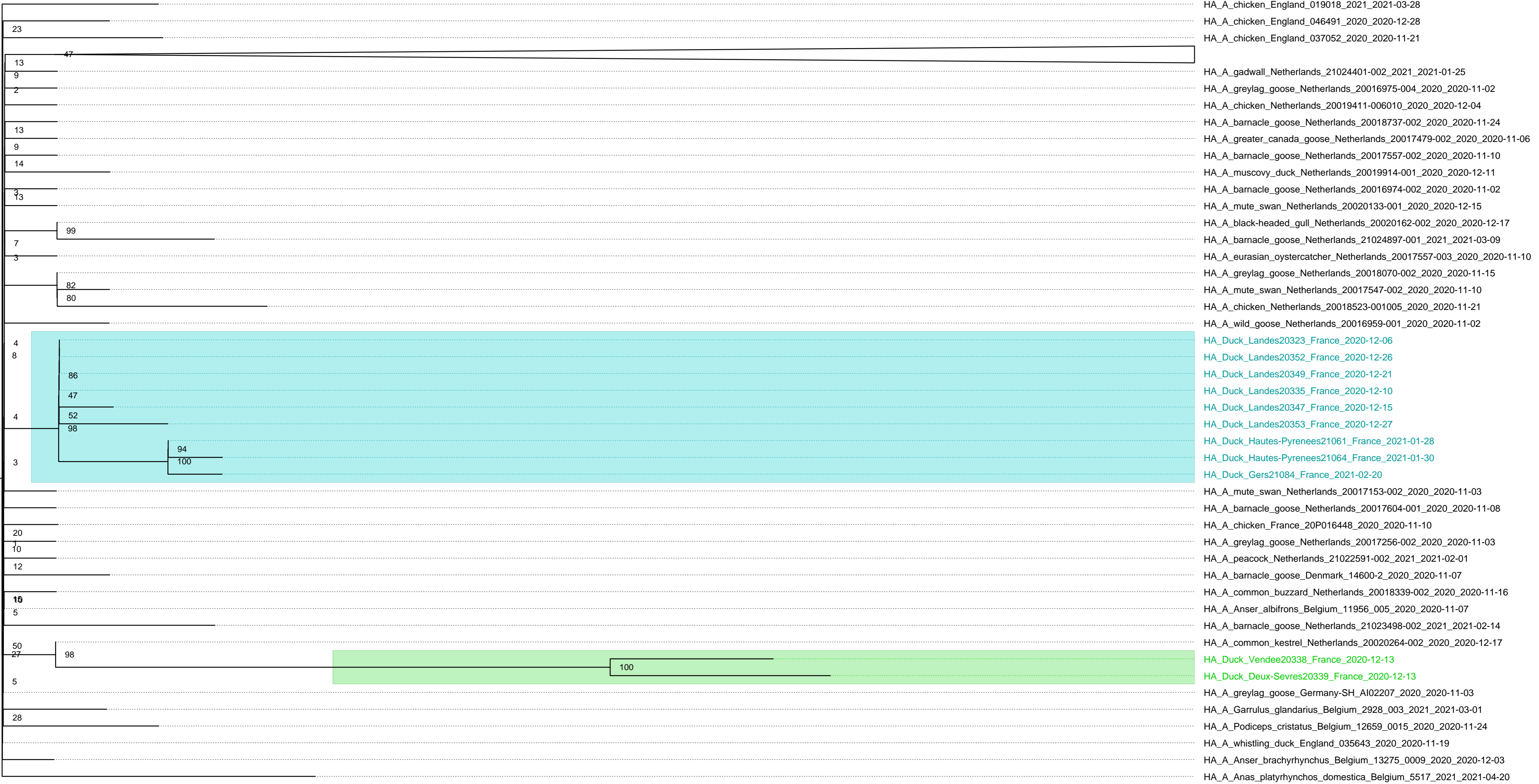

0.002

Supplement: Supplementary file 6 [file DataSheet_2.pdf]

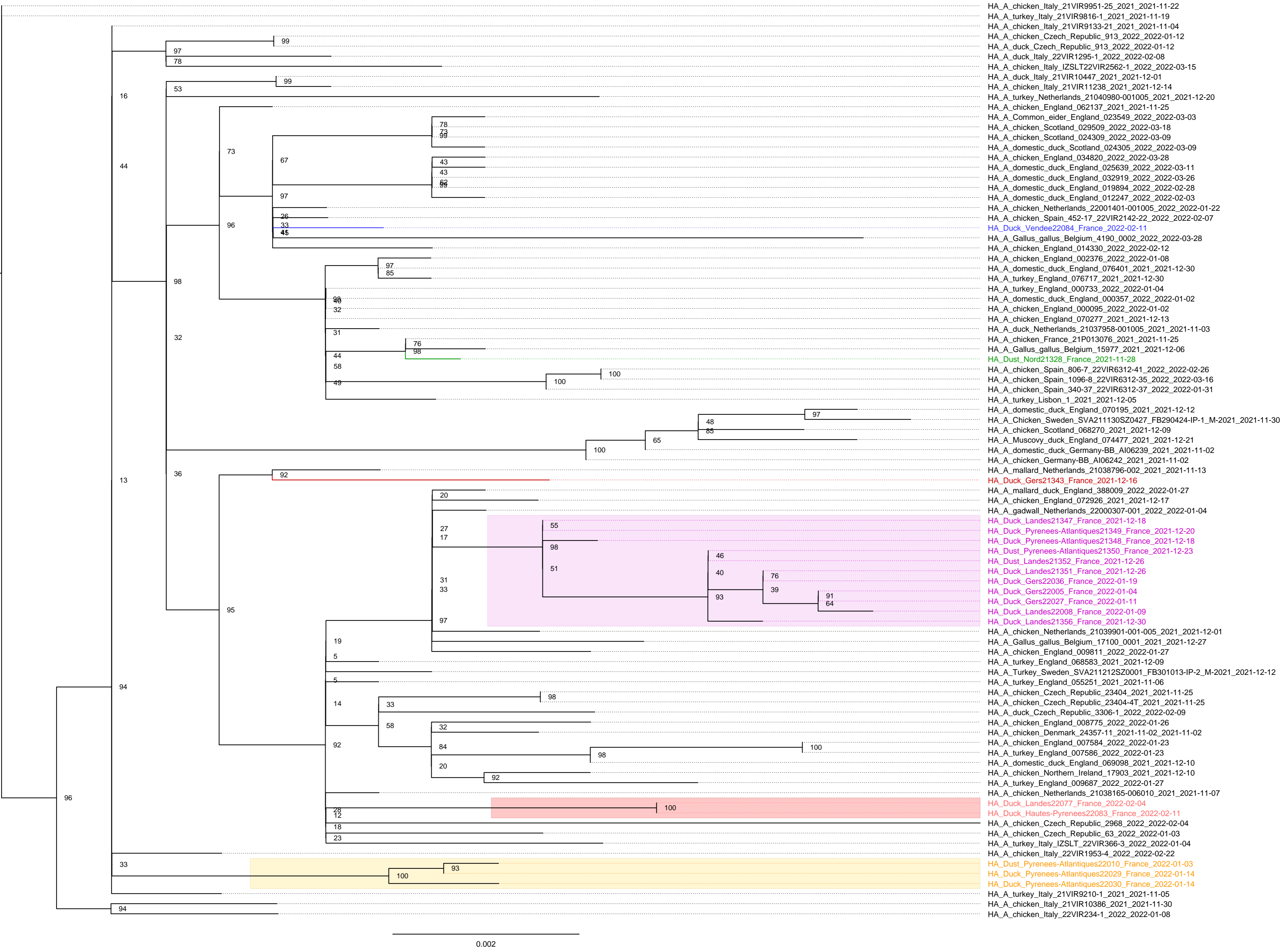

Supplement: Supplementary file 7 [file DataSheet_3.pdf]

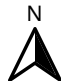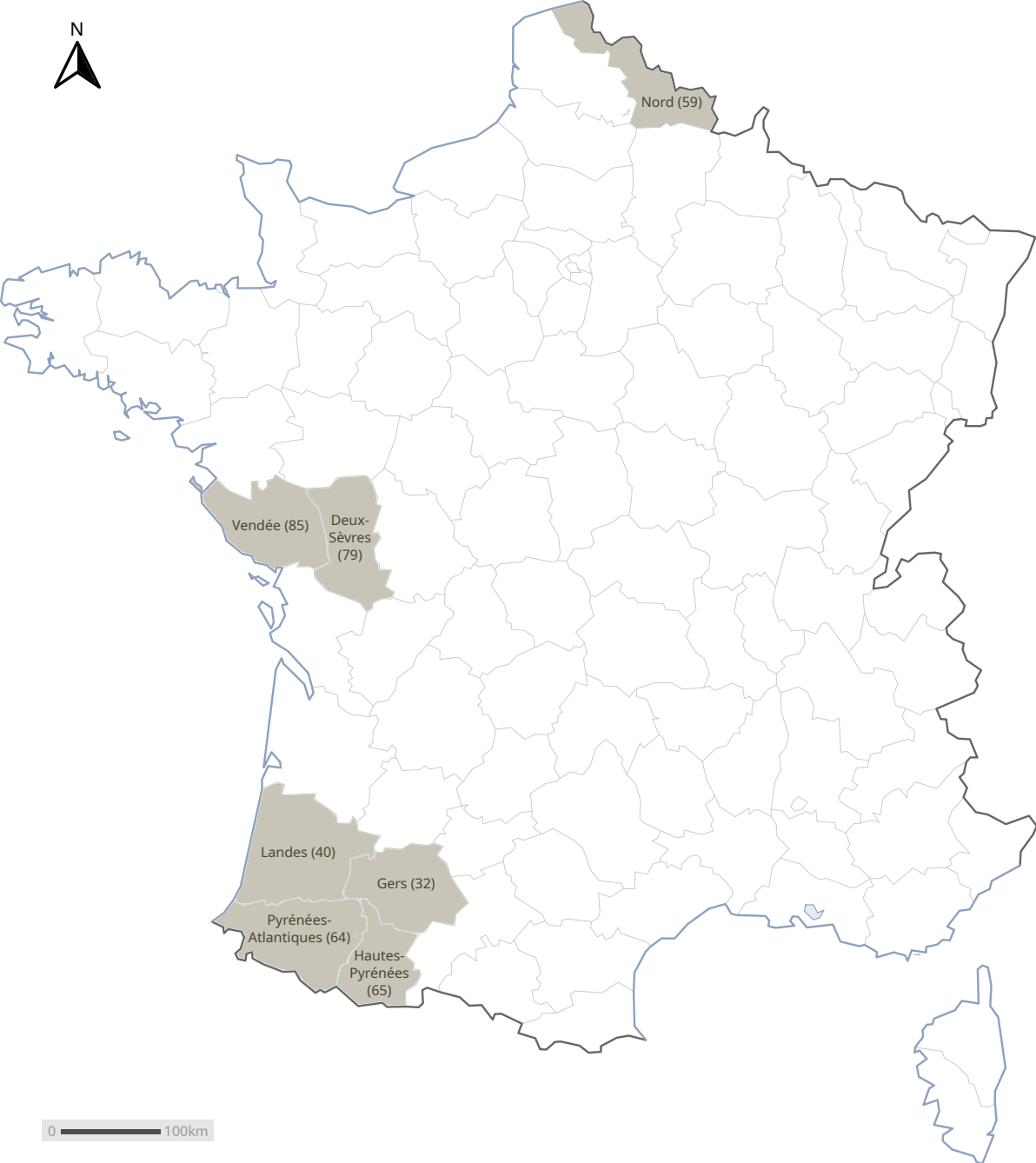

0 100km

Supplement: Supplementary file 8 [file DataSheet_4.pdf]
