# Supplementary material for: An amplicon-based nanopore sequencing workflow for rapid tracking of avian influenza outbreaks, France, 2020-2022
Source: Front Cell Infect Microbiol. 2024 Jan 22;14:1257586. doi: 10.3389/fcimb.2024.1257586 (PMC10839014; doi:10.3389/fcimb.2024.1257586)
Supplement: Supplementary file 5 [file DataSheet_1.pdf]

Supplementary_file_1_2020-2022 Outbreaks sequencing

Reference	Sample type	Sampling date (YYYY-MM-DD)	Host	French department	RTqPCR CT value (M gene)	Total yield (Mb)	N50 (bp)	HA accession number	HA yield (Mb)	N50 HA (bp)	10X_HA
20323	Tracheal swab	2020-12-06	Mule_duck	40	28,5	230,59	640	MZ166252	30,53	661	100,0%
20335	Tracheal swab	2020-12-10	Mule_duck	40	24,7	139,52	891	MZ166260	6,05	636	100,0%
20338	Tracheal swab	2020-12-13	Mule_duck	85	27,1	96,09	572	MZ166268	0,36	469	100,0%
20339	Dust	2020-12-13	Mule_duck	79	20,4	129,52	882	MZ166276	9,10	641	100,0%
20347	Tracheal swab	2020-12-15	Mule_duck	40	14	474,13	937	MZ166239	45,34	1 818	100,0%
20349	Tracheal swab	2020-12-21	Mule_duck	40	20,4	2,34	896	MZ166284	0,17	656	100,0%
20352	Tracheal swab	2020-12-26	Mule_duck	40	18,9	289,83	808	MZ166292	22,99	648	100,0%
20353	Feather	2020-12-27	Mule_duck	40	23,5	301,95	897	MZ166300	28,36	598	100,0%
21061	Dust	2021-01-28	Mule_duck	65	25	28,62	443	MZ166240	1,17	681	100,0%
21064	Tracheal swab	2021-01-30	Mule_duck	65	20	28,35	872	MZ166308	7,65	652	100,0%
21084	Tracheal swab	2021-02-20	Mule_duck	32	22,8	366,39	888	MZ166316	32,68	673	100,0%
21328	Dust	2021-11-28	Chicken	59	26,23	88,81	913	OQ632895	81,2	915	100,0%
21343	Feather	2021-12-16	Mule_duck	32	16,02	65,13	881	OQ632818	30,24	654	100,0%
21347	Feather	2021-12-18	Mule_duck	40	16,21	153,82	713	OQ632819	79,53	658	100,0%
21348	Feather	2021-12-18	Mule_duck	64	13,03	205,89	682	OQ632820	121,51	611	100,0%
21349	Feather	2021-12-20	Mule_duck	64	13,23	107,84	663	OQ632821	62,74	615	100,0%
21350	Dust	2021-12-23	Mule_duck	64	21,39	76,67	905	OQ632822	12,81	731	100,0%
21351	Feather	2021-12-26	Mule_duck	40	18,14	76,03	883	OQ632823	33,46	646	92,5%
21352	Dust	2021-12-26	Mule_duck	40	23,79	87,02	616	OQ632824	14,78	660	61,6%
21356	Feather	2021-12-30	Mule_duck	40	17,56	99,36	890	OQ632825	45,56	664	100,0%
22005	Tracheal swab	2022-01-04	Pekin_duck	32	17,97	83,93	903	OQ632826	33,8	667	100,0%
22008	Feather	2022-01-09	Mule_duck	40	18,76	86,05	765	OQ632827	40,5	661	100,0%
22010	Dust	2022-01-03	Mule_duck	64	26,24	71,57	738	OQ632828	15,66	662	98,2%
22027	Feather	2022-01-11	Mule_duck	32	18,34	98,32	887	OQ632829	48,77	664	100,0%
22029	Feather	2022-01-14	Mule_duck	64	17,12	58,08	875	OQ632830	29,09	648	100,0%
22030	Feather	2022-01-14	Mule_duck	64	18,33	70,17	721	OQ632831	36,26	648	100,0%
22036	Feather	2022-01-19	Mule_duck	32	20,27	72,18	738	OQ632832	32,74	655	100,0%
22077	Cloacal swab	2022-02-04	Pekin_duck	40	27,71	78,84	630	OQ632833	11,39	658	100,0%
22083	Tracheal swab	2022-02-11	Mule_duck	65	24,69	79,12	646	OQ632834	6,6	656	100,0%
22084	Feather	2022-02-11	Turkey	85	21,02	49,45	894	OQ632835	18,42	652	100,0%
