# Supplementary material for: An amplicon-based nanopore sequencing workflow for rapid tracking of avian influenza outbreaks, France, 2020-2022
Source: Front Cell Infect Microbiol. 2024 Jan 22;14:1257586. doi: 10.3389/fcimb.2024.1257586 (PMC10839014; doi:10.3389/fcimb.2024.1257586)
Supplement: Supplementary file 9 [file DataSheet_5.docx]

**Supplementary File Image 1.** **Amplicons profile on a 1% agarose gel.** The resulting PCR products obtained from two H5N8 samples were loaded on an agarose gel. The DNA bands show the amplification of the HA with the HA primers pool in several parts (A, B), as well as the whole genome amplification with the universal primers (C,D).

**Supplementary File Image 2. Sequencing metrics and genome coverage.** Fig. 2A shows the cumulative output of bases produced as a function of time (hours). Fig. 2B shows a read length histogram with several peaks corresponding to the size of the majority amplicons. Fig. 2C shows the bases count per barcode in a sequencing run where 4 samples were multiplexed. Fig. 2D shows the coverage and sequencing depth (logarithmic scale) all over the whole genome obtained for one sample.

**Supplementary File Image 3. Results of statistical analysis showing Ct value as a function of sample type.** Comparison of the sample type and the Ct value of the M gene qPCR. Compared with dust and tracheal swabs, feathers are the type of sample with significantly lower Ct values.

**Supplementary File Image 4. Results of statistical analysis showing the percentage of data aligning with the HA segment as a function of sample type.** Comparison of sample type and proportion of sequencing data belonging to the HA gene. Feathers are the sample type with significantly higher HA data.

**Supplementary File Table 1. Accession numbers.** This file contains the GenBank accession numbers corresponding to the sequences obtained in this study.

**Supplementary File Table 2. GISAID reference sequences.** This file contains the two sets of sequences (2020-21 H5N8 and 2021-22 H5N1) used for the phylogeny analysis. The sequences are available on the GISAID EpiFlu™ Database.

**Supplementary File Data sheet 1. Details on outbreaks sequencing.** This file includes several data such as (i) the laboratory reference number corresponding to the samples sequenced, (ii) the sample type, (iii) the sampling date (iv) the host species sampled, (v) the French department where the farms were located, (vi) the M gene RTqPCR CT value obtained, (vii) the quantity of bases obtained with nanopore sequencing for the sample, (viii) the median size of the reads generated with nanopore sequencing, (ix) the accession number corresponding to the hemagglutinin gene, (x) the quantity of bases with nanopore sequencing corresponding the hemagglutinin gene of the sample, (xi) the median size of the reads generated with nanopore sequencing corresponding to the hemagglutinin gene, (xii) the proportion of HA sequenced at least ten times.

**Supplementary File Data sheet 2. Phylogenetic tree, 2020-21 H5N8 AIV outbreaks.** Phylogenetic tree showing the relationship between nine outbreaks (blue taxa) in southwest France sampled between 6 December 2020 and 20 February 2021. In addition, at a distance of about 300 km (in Vendée and Deux-Sèvres), two other outbreaks (green taxa) were sampled on 13 December 2020. These results assume that there is no relationship between the two groups of analyzed HPAIV outbreaks. The values on the branches are the bootstrap percentages and the scale bar represents the expected number of nucleotide substitutions per site.

**Supplementary File Data sheet 3. Phylogenetic tree, 2021-22 H5N1 AIV outbreaks.** Phylogenetic tree showing 19 outbreaks clustering in six different groups. These results suggest the circulation of one viral strain (pink taxa) in southwest France between 11 farms (between 18 December 2021 to 19 January 2022). In the same area six outbreaks were analyzed (yellow, light red, dark red taxa) and group in three different clusters, these results assume that there is no relationship between the four groups of HPAIV outbreaks described above. The values on the branches are the bootstrap percentages and the scale bar represents the expected number of nucleotide substitutions per site.
